# Molecular Insights Into Hypervirulent *Klebsiella pneumoniae*: Identification and Correlation of Virulence Determinant

**DOI:** 10.1002/mbo3.70433

**Published:** 2026-10-14

**Authors:** Fathy M. Elkady, Nosiba S. Basher, Mohammed S. Abdulrahman, Amr H. Hashem, Rafik Abdellatif Metwally, Ahmed Abdel Tawab, Ebrahim Saied, Nasir A. Ibrahim, Mohammed Aufy

**Affiliations:** ^1^ Microbiology and Immunology Department Faculty of Pharmacy (Boys) Al‐Azhar University Cairo Egypt; ^2^ Department of Biology College of Science Imam Mohammad Ibn Saud Islamic University (IMSIU) Riyadh Saudi Arabia; ^3^ Department of Microbiology and Immunology Faculty of Pharmacy Menoufia National University Menoufi Egypt; ^4^ Department of Botany and Microbiology Faculty of Science Al–Azhar University Cairo Egypt; ^5^ Department of Clinical Pathology Faculty of Medicine Al‐Azhar University Cairo Egypt; ^6^ Department of Microbiology and Immunology, Faculty of Medicine Al–Azhar University Cairo Egypt; ^7^ Department of Pharmaceutical Sciences, Division of Pharmacology and Toxicology University of Vienna Vienna Austria

**Keywords:** biomarkers, hypervirulent, *K. pneumoniae*, mucoid regulator, siderophores, string test

## Abstract

*Klebsiella pneumoniae* is an increasingly important opportunistic pathogen worldwide, particularly due to the emergence and spread of hypervirulent *K. pneumoniae* (hvKP), a clinically significant pathotype first identified in Taiwan. This study aimed to molecularly identify hvKP isolates screened for coexisting virulence determinants and evaluate the association between specific virulence genes and hypervirulence. Clinical specimens were collected from hospitalized patients, and *K. pneumoniae* isolates were identified phenotypically using microscopic culture characteristics and biochemical assays and genotypically confirmed by PCR targeting the *khe* gene. Hypermucoviscosity regulators _p_ *rm*pA and _p_ *rmp*A2, siderophore‐encoding genes *iuc*A and *iro*N, and other virulence‐associated genes including *wzi*, *mag*A, *wzy*K2, *wab*G, and *peg‐*344 were investigated. Eighty‐two capsulated non‐motile *K. pneumoniae* isolates were confirmed with _p_ *rm*pA and _p_ *rmp*A2 detected in 42 and 29 isolates, respectively, whereas *iuc*A was identified in 31 isolates and *iro*N in 6 isolates only. Based on the molecular criteria, 34 isolates were classified as hvKP and 48 as classical *K. pneumoniae* (cKP). A strong association was observed between the *peg‐*344 gene and hypervirulence (*p* = 0.0001), whereas hypermucoviscosity showed a weaker correlation. However, low‐speed sedimentation assay (LSSA) analysis demonstrated a significant relationship between the hypermucoviscous phenotype and genotypic hypervirulence (*p* = 0.041). These findings highlight the molecular determinants of hvKP and identify *peg*‐344 as the gene most strongly associated with hypervirulence; however, its moderate sensitivity (58.8%) and absence in over 40% of hvKP isolates indicate that it is better suited as a supportive confirmatory marker than a standalone diagnostic biomarker for hvKP

## Graphical abstract

Genotypic characterization of 82 clinical *K. pneumoniae* isolates identified 34 hypervirulent strains. The *peg‐344* most strongly associated with hypervirulence (*p* = 0.0001) but with only 58.8% sensitivity and no signal in over 40% of hvKP isolates, it's a confirmatory marker, not a standalone diagnostic.

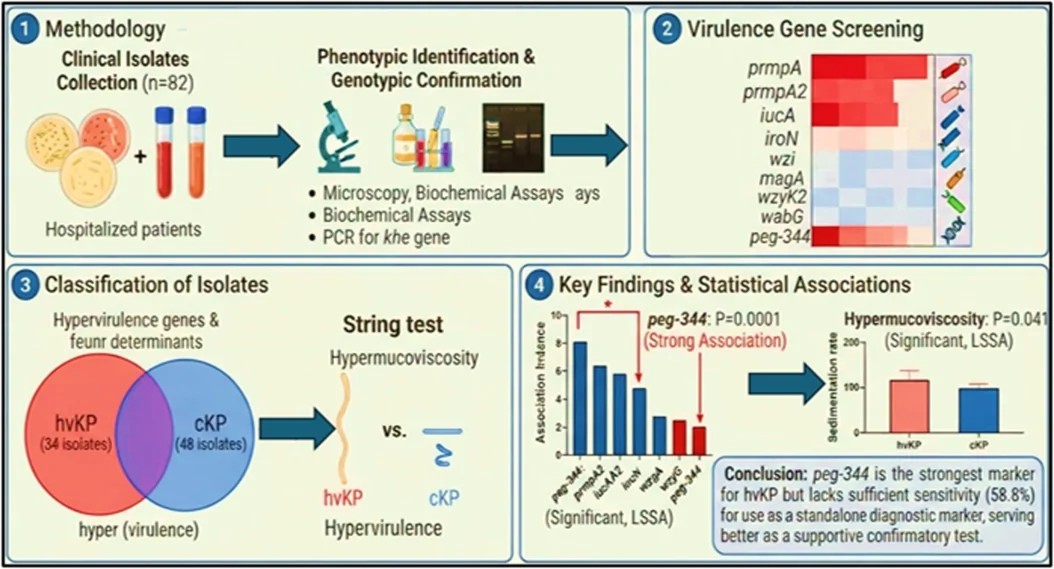

## 1 Introduction

The distinctive hvKP strain, which is associated with increased morbidity and mortality rates, was first observed in Taiwan and has since been recorded worldwide (Parrott et al. 2021; Zhu et al. 2021; Ouyang et al. 2022). These invasive strains are characterized by their tendency for metastatic spreading. Subsequently, they have been associated with a high ability to cause serious community‐acquired bacterial infections, including pneumonia with simultaneous meningitis, endophthalmitis, and pyogenic liver abscess (Whitaker et al. 2018; Chang et al. 2021).

The hvKP pathotype is usually associated with two groups of factors, namely mucoid regulators and siderophores (Kochan et al. 2023). The plasmid‐encoded regulator of mucoid phenotype A (_p_ *rmp*A/A2) genes represents the organizers of the extracellular capsular polysaccharide synthesis and subsequently the hypermucoviscous (hmv) phenotype regulator. These genes carried on a large (~200 kb) typical virulence plasmid, pLVPK, control the capsule consistency and are closely associated with the hmv colony appearance (Compain et al. 2014; Ballén et al. 2021). Significantly, the hmv has been used synonymously to describe the hvKP strains. However, it was recently established that not all hmvKP strains resulted in metastatic infection, the distinctive hmv phenotype was not observed in all hvKP strains, and the hmv phenotype was also detected in some cKP isolates (Mendes et al. 2023).

On the other hand, 4 siderophores iron scavengers, namely aerobactin, salmochelin, enterobactin, and yersiniabactin, allow the bacterial cells to obtain their iron requirements, which are essential for metabolism and infection, from the host cell (Saddam et al. 2023). Siderophore gene clusters represent essential contributors for hvKP (Elbrolosy et al. 2021). Aerobactin represents the essential siderophore for *K. pneumoniae* pathogenicity; it increases strain virulence by 100‐fold in mouse lethality tests (Jian‐Li et al. 2017). Thus, occasionally hvKP strains identification depends on the detection of genes encoding for aerobactin siderophore biosynthesis, namely *iuc*A (Alharbi et al. 2023). Also, the gene encoding for salmochelin (*iro*) biosynthesis is regularly detected in hvKP strains (Kochan et al. 2023).

Additionally, the phenotypic hvKP strains were preliminarily differentiated from the cKP strains depending on their siderophore production of more than 30 μg/mL. However, its genotypic characterization is based on the positivity of only one virulent indicator that confers the hypervirulent pathotype. These biomarkers include _p_ *rmp*A, _p_ *rmp*A2, putative metabolite transporter (*peg*‐344), *iro*B associated with salmochelin siderophore biosynthesis, and *iuc*A genes (Hefzy et al. 2023; Mai et al. 2023). Also, the presence of genetic biomarkers, including *iuc*A and _p_ *rmp*A genes, has been considered to distinguish between hvKP and cKP strains (Ballén et al. 2021). Moreover, recent sequencing of the hvKP genome revealed the prospective relationship between these biomarkers and the mucoviscosity‐associated gene A (*mag*A), the gene encode capsular serotype K1, and the hvKP pathotype (Neumann et al. 2023). Furthermore, the *wzi* virulence determinant conserved in all capsular serotypes encodes an outer membrane protein that is responsible for host cell surface attachment and implicated in the anchoring of *K. pneumoniae* capsular polysaccharides (Neumann et al. 2023). To date, at least 77 capsular serotypes (K1‐K77) have been recognized in *K. pneumoniae* (Saddam et al. 2023). Characteristically, serotypes K1 and K2 are described as possible causes of severe human infections and are closely associated with the hvKP pathotype (Jian‐Li et al. 2017; Guo et al. 2023; Dong et al. 2024). Furthermore, hvKP strains' colonization, invasion, and pathogenesis are also concurrent with the *wab*G virulent gene accountable for capsular lipopolysaccharide production (Izquierdo et al. 2003). Accordingly, the current unified biomarker or exact criteria for accurate distinction between the hvKP and cKP strains remain unrecognized (Guo et al. 2023; Kochan et al. 2023; Mendes et al. 2023).

From the previous background, our research aimed to molecular screening for the coexistence of some genetic biomarkers associated with the hvKP pathotype amongst *K. pneumoniae* clinical isolates. Also, we aimed to find possible correlations between hvKP pathotypes and other virulent genes. Additionally, we comparatively evaluated the capability of two phenotypic methods routinely employed for the detection of hvKP strains.

## 2 Materials and Methods

### 2.1 The Tested Isolates

Under aseptic conditions, sterile containers were used to collect 428 non‐duplicate clinical specimens from patients with bacteriological work‐up attending different departments at Sayed Galal hospital, Al‐Azhar University, Cairo, Egypt, between May 2025 to December 2025. The inclusion criteria for the selected specimens included collection from hospitalized patients with clinical suspicion of bacterial infection and yielded pure, phenotypically identified *K. pneumoniae* growth. Specimens were excluded if they represented duplicate isolates from the same patient and infection episode, were contaminated or showed mixed bacterial growth precluding isolation of a pure culture, or originated from patients with incomplete clinical and/or specimen documentation. The clinical specimens, depending on the patient's clinical manifestation, were sputum (103), blood (72), pus (87), urine (83), ear discharge (72), and vaginal swabs (11). The bases described by Whitaker et al. (2018) and the guidelines of Procop et al. (2020) were followed for initial identification of *K. pneumoniae*. Briefly, microscopical criteria, including Gram reaction, capsule formation, and motility, were examined. Also, the phenotypic culture properties were assessed after growth on the MacConkey and triple sugar iron agar (TSI) (Fatsis‐Kavalopoulos et al. 2024). Biochemically, citrate consumption, indole production, methyl red, and Voges‐Proskauer tests were also performed. Additionally, the capability for oxidase, catalase, and urease enzymes production was evaluated. On the molecular side, total bacterial DNA from phenotypically considered *K. pneumoniae* isolates was obtained using a boiling procedure (Elkady et al. 2024). Afterward, the isolates were genotypically identified via the PCR amplification technique using specific primers (Table 1) that target the *khe* characteristic gene (Maleki et al. 2023). For each isolate, the amplification blend contained Cosmo PCR Red Master Mix (10 μL) (Willowfort, Birmingham, UK), 10 pmol forward and reverse primers (1 μL each), extracted DNA (2 μL), and nuclease‐free water (6 μL). Positive and negative control experiments were performed using DNA from *K. pneumoniae* ATCC13882 standard strain and nuclease‐free water, respectively. A thermal cycler with a suitable program (Table 2) was employed for amplification of each gene sequence (Biometra UNO–Thermoblock, Germany). Separation of PCR products was performed by 45 min of electrophoresis at 100 V using 1.5% agarose gel stained with 0.05 mg/ml ethidium bromide in the presence of Tris‐acetate EDTA electrophoresis buffer (Sigma‐Aldrich, Hamburg, Germany). Finally, the amplified products were observed by the aid of a UV transilluminator (HERMLE Labortechnik GmbH, Wehingen, Germany), and a 1500 bp DNA ladder (Cat. No. MD1019) (Hangzhou Simgen Biotechnology Co., Ltd., China) was used for each PCR amplification fragment‐specific size determination (Elkady et al. 2022).

**Table 1:** Oligonucleotide sequences target particular genes in the tested *K. pneumoniae* isolates.

Genes	Sequence of‐specific primer (5′ 3′)	Size of amplicon (bp*)	Reference
*khe*	F*; TGATTGCATTCGCCACTGG	428	Maleki et al. (2023)
	R*; GGTCAACCCAACGATCCTG		
_p_ *rmp*A	F; ATGTGGCTTGACGTTTCGGGGG	160	Wei et al. (2022)
	R; GCCGTGGATAATGGTTTACAATTCGGC		
_p_ *rmp*A2	F: GGATGTGGCTTGACATTTCGGGGG	227	
	R; TTCATGGATGCCCTCCCTCCTG		
*iuc*A	F; GCTTATTTCTCCCCAACCC	583	
	R; TCAGCCCTTTAGCGACAAG		
*iro*N	F; CCGCAAAGAGACGAACCGCCTT	546	
	R; CGGGCAATCCCCGCTTTGACTT		
*peg‐*344	F; AAAGGACAGAAAGCCAGTG	411	
	R; CAATGACGAGGGGGATAATC		
*wzi*	F; GTGCCGCGAGCGCTTTCTATCTTGGTATTCC	580	Brisse et al. (2013)
	R; GAGAGCCACTGGTTCCAGAA[C or T]TT[C orG] ACCGC		
*wab*G	F; ACCATCGGCCATTTGATAGA	683	Anis et al. (2021)
	R; CGGACTGGCAGATCCATATC		
*the magA*(*wzy*K1)	F; GGTGCTCTTTACATCATTGC	1282	
	R; GCAATGGCCATTTGCGTTAG		
*wzy*K2	F; GACCCGATATTCATACTTGACAGAG	641	Ssekatawa et al. (2021)
	R; CCTGAAGTAAAATCGTAAATAGATGGC		

*F: forward, R: reverse, and bp: base pair.

**Table 2:** Specific programs for PCR amplification of each target gene.

Target genes	PCR amplification conditions			Reference
	Initial denaturation	Elongation	Final extension	
*khe*	94°C/5 min	35 cycles of: 94°C/45 s, 55°C/40 s, and 72°C/60 s	72°C/10 min	Maleki et al. (2023)
_p_ *rmp*A_p_ *rmp*A2*iro*N	95°C/5 min	35 cycles of: 95°C/30 s, 58°C/60 s, and 72°C/60 s		Wei et al. (2022)
*iucA*		25 cycles of: 95°C/30 s, 59°C/30 s, and 72°C/50 s		
*peg‐*344		25 cycles of: 95°C/30 s, 53°C/30 s, and 72°C/30 s		
*wzi*	94°C/5 min	30 cycles of: 94°C/30 s, 55°C/40 s, and 72°C/30 s		Brisse et al. (2013)
*wab*G(*mag*A)	95°C/5 min	30 cycles of: 95°C/60 s, 60°C/30 s, and 72°C/60 s		Anis et al. (2021)
*wzy*K2		30 cycles of: 95°C/30 s, 50°C/30 s, and 72°C/30 s		Ssekatawa et al. (2021)

### 2.2 Molecular Detection of Hypervirulent‐Related Determinants

The presence of hypervirulent extremely related virulent biomarkers was evaluated in the tested *K. pneumoniae* clinical isolates to identify hvKP strains. They were screened for _p_ *rmp*A and _p_ *rmp*A2 genes (Martin et al. 2018) and siderophore‐mediated iron acquisition‐associated genes, including *iuc*A and *iro*N, which encode salmochelin (Yu et al. 2018). Also, the PCR technique was used to assess the existence of the *wzi* gene (Brisse et al. 2013) as well as the capsular serotypes K1 and K2 encoding genes, *mag*A and *Wzy*K2, respectively (Ssekatawa et al. 2021). Additionally, the occurrence of other probably hypervirulent‐associated genes, including the *wab*G biofilm formation‐associated gene (Anis et al. 2021) and the *peg*‐344 gene (Parrott et al. 2021), was examined. The PCR reaction mixture consisted of the tested bacterial DNA (2 µL), Cosmo PCR Red Master Mix (10 µL) (Willowfort, UK), 10 pmol primers (1 μL each) specific for each target gene amplification (Table 1), and 10 μL nuclease‐free water to a final volume of 25 µL. Each reaction program, conducted in a Thermal cycler (Biometra UNO–Thermoblock, Germany), was adjusted under the amplification conditions appropriate for each gene (Table 2). The PCR products were then separated by electrophoresis using an ethidium bromide‐stained 1.5% agarose gel, observed by the aid of a UV transilluminator, and its specific size was determined by comparing with a 1500 bp DNA ladder (Elkady et al. 2022).

Due to the absence of a clear combination of phenotypic and genotypic markers with mostly accurate prediction of the hypervirulent phenotype, the association of at least two of the assayed hypervirulent particularly correlated genes, one hmv‐related gene (_p_ *rmp*A or _p_ *rmp*A2) and one siderophores‐encoding gene (*iuc*A or *iro*N), was considered as a biomarker for their feasible genotypic hypervirulent character. While a universal consensus on the molecular definition of hvKP is still evolving, this combinatory approach is grounded in established genomic frameworks. The coexistence of these two categories of virulence factors—capsular regulation and enhanced iron acquisition—has been demonstrated as highly accurate (up to 94%) for predicting the hypervirulent pathotype and distinguishing it from classical strains (Russo and Marr 2019; Stanton and Wyres 2024). By requiring markers from both functional groups, we aimed to ensure a robust classification that aligns with the current understanding of hvKP pathogenesis, which typically involves both hypermucoviscosity and superior fitness in iron‐limited host environments (Choby et al. 2020).

### 2.3 Assessment of Mucoviscosity

The phenotypically hmvKP isolates were detected via string test and LSSA using overnight culture at 37°C of mucoid bacterial colonies on a blood agar plate. In a string test, the expansibility of a viscous string from the tested colony was assessed. A positive result was dependent on the ability of the bacteriological loop to create a thick string ≥ 5 mm (Rastegar et al. 2019). In the LSSA described by Gálvez‐Silva et al. (2024), appropriate bacterial colonies were suspended in sterile phosphate‐buffered saline and diluted to 5 mL. Each tested bacterial suspension was then adjusted to an optical density (OD) of 4 at 600 nm (OD_600_ = 4) and transferred into a sterile 15 mL conical tube. After 5 min of centrifugation at 1000 *g*, the supernatant OD_600_ was measured. A higher supernatant OD_600_, in the case of hmvKP, close to that value before centrifugation than that of cKP was expected, as the tested hmv strains tended to remain in the supernatant due to their elevated capsule production that subsequently prevented the tight pellet formation by hmvKP strains.

### 2.4 Data Integration and Statistical Analysis

In this research, the mean ± SD was used to present the results of the experiments performed as triplicate. Also, the relative frequencies and percentages were used as descriptive data. Additionally, sensitivity, specificity, accuracy, positive and negative predictive values, and Cohen's kappa (*κ*) statistics were calculated with 95% confidence intervals (Wilson score method for sensitivity, specificity, accuracy, positive predictive value (PPV), and NPV = negative predictive value (NPV); standard asymptotic method for *κ*). For 2 × 2 contingency tables in which any expected cell count was below 5, Fisher's exact test was used in place of the chi‐square test. One‐way ANOVA was used to compare the mean triplicate OD_600_ measurements obtained in the low‐speed sedimentation assay among hvKP, cKP, and control isolates.

## 3 Results and Discussions

### 3.1 *K. pneumoniae* Isolates

A total of 82 oxidase‐negative, non‐motile, capsulated, Gram‐negative rods that were able to ferment lactose sugar in MacConkey's agar and produce large mucoid colonies were initially predicted as *K. pneumoniae*. A negative methyl red test, failure for indole production, and positive ability for urease, citratase, and catalase enzymes production established their phenotypic recognition. Also, the TSI growth showed H_2_ gas, acidic slant and butt, and no H_2_S gas formation were biochemically confirmed their identification. On the molecular side, findings of the characteristic *khe* gene‐specific band, at about 428 bp (Figure 1), indicate genotypic confirmatory identification of the obtained isolates. These isolates were obtained from sputum (23; 28%), blood (11; 13.5%), pus (19; 23.2%), urine (23; 28%), ear discharge (5; 6.1%), and vaginal swab (1; 1.2%). Statistically significant variations in the distribution of *K. pneumoniae* isolates were recorded across the different types of clinical specimens (*χ* ^2^ = 32.56, *df* = 5, *p* < 0.05). Higher numbers in sputum, urine, and pus and very low numbers in vaginal swabs suggest clinical relevance or route of infection.

**Figure 1:**
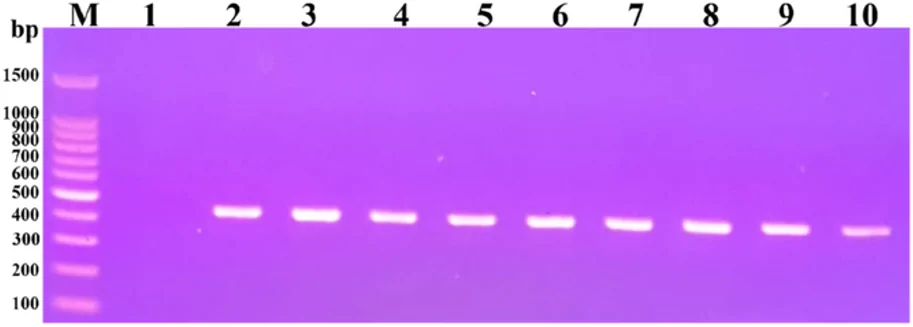
Agarose gel electrophoresis showing PCR amplicons of the *khe* gene. Lane M: 1500 bp DNA ladder; lane 1: negative control test; lane 2: *khe* gene‐specific band (about 428 bp) using DNA of *K. pneumoniae* ATCC13882 standard strain as a positive control; lanes 3‐10: representative *khe* gene‐specific bands using DNA of phenotypically detected *K. pneumoniae* isolates 1‐8, respectively.

### 3.2 Hypervirulent‐Related Determinants

The hvKP strains have been increasingly recorded globally, with the ability to cause metastatic spread combined with life‐threatening infections, including liver abscess, multifocal pneumonia, perisplenic abscess, perigastric abscess, tricuspid valve infective endocarditis, and septic emboli (Ombada et al. 2023). Unfortunately, no clear biomarkers correlated to specific virulence factors can appropriately decide the hvKP identification (Guo et al. 2023). The commonly identified key virulence elements of hvKP included iron acquisition siderophore systems, capsular overproduction, colibactin toxin, and hypermucoviscosity (Chen et al. 2023). In our study, the PCR assays' findings are represented in Figure 2. The tested *K. pneumoniae* isolates demonstrated relatively high frequencies of _p_ *rmp*A and _p_ *rmp*A2 genes at 42/82 (51.2%) and 29/82 (35.4%), respectively. Additionally, at least one of these two genes was present in 47/82 (57.3%) of the tested isolates. On the other hand, the *iuc*A gene was observed in 31/82 (37.8%) of the tested isolates, while the *iro*N gene had low existence, 6/82 (7.3%). Interestingly, out of the 36 isolates with at least one siderophore‐encoding gene (36/82; 43.9%), 34 isolates (34/82; 41.5%) were carried at least one of the _p_ *rmp*A and _p_ *rmp*A2 genes. Accordingly, these 34/82 (41.5%) *K. pneumoniae* clinical isolates were considered genotypically hypervirulent, while the remaining 48/82 (58.5%) *K. pneumoniae* isolates were recorded as a classical genotype. As expected, given the classification criterion, isolates designated as hvKP carried at least one of _p_ *rm*pA/_p_ *rm*pA2 and at least one of *iuc*A/*iro*N by definition; the prevalence of each individual gene among hvKP versus cKP isolates.

**Figure 2:**
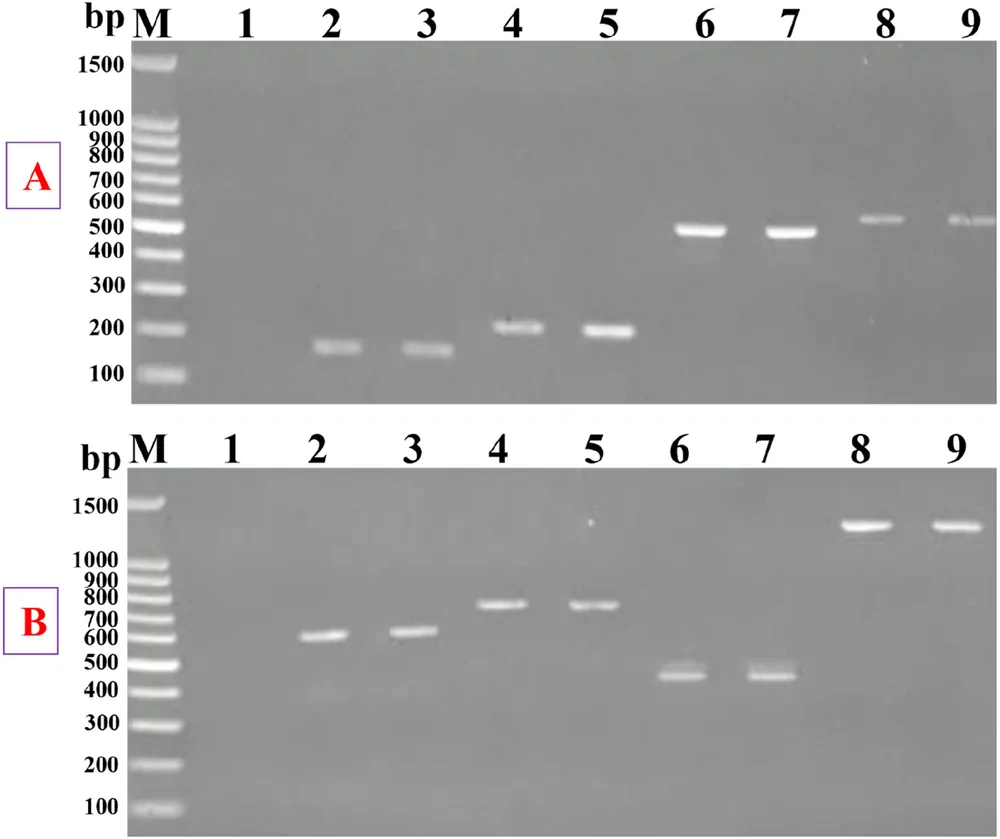
Representative agarose gel of PCR amplicons for different genes. Lanes; M: 1500 bp DNA ladder, 1: negative control, and 2‐9: target genes specific bands. (A) lanes 2‐3: *rmp*A, lanes 4‐5: *rmp*A2, lanes 6‐7: *iro*N, and lanes 8‐9: *iuc*A. (B) lanes 2‐3: *wzi*, lanes 4‐5: *wab*G, lanes 6‐7: *peg*344 gene, and lanes 8‐9: *mag*A.

In a related study in the USA (McElheny et al. 2025), 273 *K. pneumoniae* isolates were analyzed based on string test and multiplex PCR screening for _p_ *rmp*A, _p_ *rmp*A2, *iut*A, and *iro*N virulence genes. Of their tested isolates, 11 (4%) were identified as positive string test *K. pneumoniae*, and only 2 (0.7%) isolates were positive for the 4 basic virulence genes and were recorded as hvKP. The remaining string tested positive, and all string test‐negative isolates did not carry any of the tested genes. Also, (Merla et al. 2024) study in Italy recorded that 13/354 (3.7%) *K. pneumoniae* clinical isolates were hypervirulent depending on their sequence type and the existence of virulence genes, including salmochelin and aerobactin. Interestingly, 6/13 (46.1%) isolates possessed _p_ *rmp*A, *iro*N, *peg*‐344, and *iut*A virulence genes; at least one of these virulence genes was detected in each isolate, and only 7/13 (53.8%) of these isolates were string test positive. Additionally, a Chinese study by (Jiang et al. 2024) identified hvKP strains at a rate of 17.9%, depending on the detection of _p_ *rmp*A, _p_ *rmp*A2, *iuc*A, *iro*B, and *peg*‐344 genes. Furthermore, the study by Matsuda et al. (2023) in Japan genotypically identified hvKP strains by a criterion based on possessing one or more hypervirulence‐related genes, including the _p_ *rmp*A, _p_ *rmp*A2, *peg*‐344, and *iuc*A. Among their tested *K. pneumoniae* (500 isolates), the _p_ *rmp*A, _p_ *rmp*A2, *peg*‐344, and *iuc*A virulence genes were identified in 111 (22.2%), 99 (19.8%), 101 (20.2%), and 105; (21%) isolates, respectively. Additionally, amongst 66 carbapenem‐resistant *K. pneumoniae* strains, the _p_ *rmp*A2, _p_ *rmp*A, *iut*A, and *iro*N virulence genes were detected in 32 (48.5%), 15 (22.7%), 32 (48.5%), and 10 (15.2%) strains, respectively. All carbapenem‐resistant hvKP (45 strains) showed positive results for these hypervirulence‐related genes (Yu et al. 2018). Moreover, the study by Sharma et al. (2024) in India considered the detection of either or both *iuc*B and _p_ *rmp*A genes for the identification of 11 hvKP strains from 29 *K. pneumoniae* clinical isolates. In another related study, PCR detection of the _p_ *rmp*A, _p_ *rmp*A2, and *iuc*A genes in 108 clinically hvKP strains revealed the presence of the _p_ *rmp*A gene in 10 strains, the _p_ *rmp*A2 gene in 47 strains, and the *iuc*A gene in 47 strains (Mai et al. 2023). Also, 50% of the tested *K. pneumoniae* strains were accurately identified as hvKP by the concurrent detection of _p_ *rmp*A, _p_ *rmp*A2, *iuc*A, *iro*B, and *peg*‐344 genes (Liu et al. 2020). Similarly, a combination of biomarkers carried on large pLVPK and pK2044 virulent plasmids, including _p_ *rmp*A and _p_ *rmp*A2 genes, and *peg*‐344, *iro*B, and *iuc*A genes were considered the most accurate method to differentiate between hvKP and cKP strains (Mendes et al. 2023). Additionally, the hvKP was defined by their combination of hypermucoviscosity, phenotypically determined by the string test, and aerobactin positivity, genotypically detected by PCR (Alharbi et al. 2023). On the other hand, the hvKP strains mostly possess a pLVPK large virulence plasmid that carries aerobactin and _p_ *rmp*A virulence genes, contributing to their phenotypic hypervirulence. As well, the _p_ *rmp*A/_p_ *rmp*A2, *iut*, *iuc*, *iro*, and yersiniabactin‐encoding genes, *ybt* and *irp*, were identified as the critical hvKP virulence determinants (Cai et al. 2022). These findings illustrate the absence of a definite target sequence and subsequent molecular detection technique for hvKP confirmation.

In the present study, the *wzi* gene was identified among *K. pneumoniae* strains at an elevated prevalence, 71/82 (86.6%). This gene was detected in 33/34 (97%) of hvKP and 38/48 (79.2%) of cKP clinical isolates. Fisher's exact test was applied; this revealed a significant association between the occurrence of *wzi* gene and the hypervirulent genotype amongst the tested strains (Fisher's exact *p* = 0.022, 95% CI: 1.06–71.48; *φ* = 0.26). In related research, the *wzi* gene was detected in all tested *K. pneumoniae* isolates (Aboulela et al. 2020). However, the *wzi* gene was highly prevalent in both the hvKP and cKP groups, which reduces its possible discriminatory power between hvKP and cKP genotypes.

Furthermore, *wzy*K2 was not observed in any tested isolate and a low existence of the *mag*A gene was observed amongst the tested *K. pneumoniae* isolates 5/82 (6.1%). Only 4/34 (11.8%) hvKP and 1/48 (2.1%) cKP clinical isolates carried the *mag*A gene. The results of statistical analysis (Fisher's exact *p* = 0.155, 95% CI: 0.67–58.79; *φ* = 0.20) illustrated the insignificant association between hypervirulence and the presence of *mag*A and *Wzy*K2 genes encoding capsular serotype K1 and K2, respectively. In a related study, all tested hvKP (19 isolates) were negative for the *mag*A gene (Song et al. 2023). However, *mag*A and *wzy*K2 genes were detected in 40% and 30% of hvKP isolates, respectively (Lee et al. 2024). Also, the *mag*A gene was detected in 11/22 (50%) of the tested hvKP strains, of which 4/11 (36.4%) strains were string test positive and 7/11 (63.6%) strains were string test negative (Devanathan et al. 2024). Additionally, the study by Wahl et al. (2024) in Germany identified hvKP strains based on the detection of *mag*A with _p_ *rmp*A and _p_ *rmp*A2 virulence‐associated genes. These findings illustrate the variable detection rates of capsular serotype K1 and K2 encoding genes amongst hvKP strains. It's recommended to study the correlation between hvKP and other wide ranges of *K. pneumoniae* capsular serotypes.

The present study also recorded the occurrence of the *peg*‐344 virulence‐correlated gene in 22/82 (26.8%) isolates, of which 20/34 (58.8%) were genotypically hvKP and 2/48 (4.2%) were cKP. These results indicated an extremely significant association (*χ* ^2^ = 27.57, df = 1, *p* < 0.0001, 95% CI: 6.82–158.23, *φ* = 0.61) between the occurrence of the *peg*‐344 gene and the hypervirulent genotype. However, as a standalone diagnostic marker, *peg*‐344 showed only moderate sensitivity (58.82%, 95% CI: 42.2%–73.6%) against the molecular hvKP classification, with 14/34 (41.2%) genotypic hvKP isolates testing *peg*‐344‐negative, despite high specificity (95.83%, 95% CI: 86.0%–98.8%) and positive predictive value (90.91%, 95% CI: 72.2–97.5%). These findings indicate that while *peg*‐344 positivity is a strong helpful confirmatory indicator of hvKP, its absence does not exclude the hypervirulent pathotype, and it should not be relied upon as a sole or standalone biomarker for hvKP screening.

In several related studies, the *peg‐*344 gene was found to be specific for hypervirulence in *K. pneumoniae*. This gene was identified in 2 *K. pneumoniae* isolates that were classified as hvKP strains depending on carrying at least two of the six assayed virulence genes, namely *iuc*A, _p_ *rmp*A, _p_ *rmp*A2, *iro*B, *mag*A, and *peg‐*344 (Qiu et al. 2024). Interestingly, the detection of the *peg*‐344 virulence‐correlated gene was sufficiently used for hvKP detection in a study by Sanikhani et al. (2021). These outcomes demonstrated that *peg*‐344 is a highly significant supportive marker for the detection of hvKP.

In terms of *wab*G virulence‐associated gene, it was observed in 74/82 (90.2%) isolates, of which 33/34 (97%) were hypervirulent and 41/48 (85.4%) were classical. The statistical results (Fisher's exact *p* = 0.131; 95% CI: 0.66–48.12; *φ* = 0.19) indicated an insignificant association between the genotypic hypervirulence and the presence of the *wab*G gene. In a similar study, the *wab*G gene was detected in 343/363 (94.5%) *K. pneumoniae*, and no correlation was found between the possession of the *wab*G gene and the detection of _p_ *rmp*A and _p_ *rmp*A2 genes (Jiang et al. 2024). These results indicated the absence of a certain correlation between the existence of the *wab*G gene and the hypervirulent character in the tested isolates of *K. pneumoniae*.

### 3.3 Phenotypic Investigation of hvKP and cKP Isolates

#### 3.3.1 String Test

Phenotypically, out of our tested 82 *K. pneumoniae* isolates, only 13 (15.8%) isolates showed positive expansibility in the string test, which specified their hmv phenotype. This observed phenotype was found in 11/34 (32.3%) hvKP and 2/48 (4.2%) cKP isolates.

Statistical investigation clarified that the string test exhibited low sensitivity (32.35%, 95% CI: 19.1%–49.2%), high specificity (95.83%, 95% CI: 86.0%–98.8%), and moderate accuracy (69.51%, 95% CI: 58.9%–78.4%); Cohen's kappa (*κ* = 0.31, 95% CI: 0.08–0.54) revealed fair agreement with molecular testing, indicating that the string test lacks sufficient reliability as a standalone diagnostic method for hvKP strains. A study conducted by Cai et al. (2022) indicated that hvKP strains usually showed a positive string test. However, they isolated a carbapenem‐resistant hvKP strain from liver abscess drainage in a patient with a liver abscess combined with multisite infections, including pneumonia, abdominal abscess, pleural infection, and splenic abscess. This strain was string test‐negative, non‐hmv, and showed high virulence and mortality rates like those of the hvKP ATCC 43816 strain. Also, Gálvez‐Silva et al. (2024) isolated a dual carbapenemase‐producing hvKP strain in Chile with an unexpected lack of hmv revealed by negative string test results. Although the wide application of hmv phenotype, determined by a semi‐qualitative string test, as a marker for hvKP identification, this assay is easily affected by the user's technique and colony conditions. Additionally, mucoviscous cKP strains and non‐hmv hvKP strains have been reported (Zhu et al. 2021). Accordingly, the string test, which is frequently used for hmv phenotype examination and consequent initial hvKP isolate detection, has limited specificity, and the cKP and hvKP strain classification cannot be definitely limited to the positivity of this test (Mai et al. 2023). These outcomes illustrate the insufficient effectiveness of the string test that is usually used as a parameter for hvKP distinguishing.

### 3.4 Low‐Speed Sedimentation Assay (LSSA)

On the other hand, 21/82 (25.6%) *K. pneumoniae* isolates showed a positive hmv phenotype based on the LSSA findings. Genotypically, this noticeable hmv phenotype was detected in 21/34 (61.8%) hypervirulent strains and was not detected in all cKP strains. Statistically, the LSSA demonstrated moderate sensitivity (61.76%, 95% CI: 45.0%–76.1%), high specificity (100%, 95% CI: 92.6%–100%), and good accuracy (84.15%, 95% CI: 74.7%–90.5%); Cohen's kappa (*κ* = 0.65, 95% CI: 0.48–0.83) indicated fair agreement with the molecular results. Related research indicated the responsibility of the large polysaccharide amount in the extended capsule of hmv *K. pneumoniae* cells for increased bacterial cell viscosity. In their study, LSSA illustrated the higher viscosity and consequent difficult precipitation of the viscous *K. pneumoniae* bacterial cells (Huang et al. 2023). However, the challenges of historical reliance on phenotypic assays like the LSSA as sole identifiers for hvKP are consistent with recent studies that highlight the complexity of hvKP pathogenesis beyond hmv phenotype alone. For instance, Capitani et al. (2024) demonstrated the emergence of hmv *K. pneumoniae* strains lacking typical hvKP virulence genes (e.g., _p_ *rmp*A‐D), acquiring the hmv phenotype through mutations in *wzi*, further supporting the notion that the hmv phenotype can be independent of conventional hypervirulence markers. Similarly, Al Ismail et al. (2025) emphasized that while the hmv phenotype is a prominent feature, not all hvKP strains consistently exhibit this phenotype, and some cKP strains can also display the hmv phenotype, underscoring the need for genotypic confirmation.

### 3.5 Comparative Analysis of Phenotypic Tests

Among the two phenotypic methods, LSSA showed comparatively better sensitivity, accuracy, and concordance with molecular testing than the string test (Table 3). However, neither method demonstrated sensitivity sufficient for standalone diagnostic use. The string test's high specificity may support confirmation of positive cases, but its very low sensitivity results in frequent false negatives. On the other hand, LSSA offers an improved diagnostic balance and perfect specificity and substantial agreement with the molecular reference standard, yet it still fails to detect over a third of true hvKP isolates. Molecular testing therefore remains essential for definitive hvKP identification, and neither phenotypic assay should be considered an adequate substitute.

**Table 3:** Summary of comparative analysis of phenotypic tests.

Metric	String test	LSSA
Sensitivity	32.35% (95% CI: 19.1–49.2%)	61.76% (95% CI: 45.0–76.1%)
Specificity	95.83% (95% CI: 86.0–98.8%)	100.00% (95% CI: 92.6–100%)
Accuracy	69.51% (95% CI: 58.9–78.4%)	84.15% (95% CI: 74.7–90.5%)
PPV	84.62% (95% CI: 57.8–95.7%)	100.00% (95% CI: 84.5–100%)
NPV	66.67% (95% CI: 54.9–76.6%)	78.69% (95% CI: 66.9–87.1%)
Kappa (κ)	0.31 (95% CI: 0.08–0.54) Fair	0.65 (95% CI: 0.48–0.83) Substantial

Abbreviations: NPV = negative predictive value, PPV = positive predictive value.

## 4 Conclusion

Our findings demonstrated that the genotypic detection of virulence markers (including _p_ *rmp*A, _p_ *rmp*A2, *iuc*A, and *iro*N) is significantly more reliable than phenotypic methods for identifying hvKP strains. While the string test and LSSA traditional phenotypic methods maintained high‐to‐perfect specificity, their limited sensitivity and (for the string test, in particular) substantial false‐negative rate rendered them inadequate as standalone diagnostic tools, although LSSA showed fair agreement with molecular testing and represented the more reliable of the two phenotypic approaches. Although *peg*‐344 demonstrated the strongest individual statistical association with the hypervirulent genotype, its moderate sensitivity and absence in a substantial proportion of hvKP isolates indicate that it should be interpreted as one component of a combinatory molecular panel rather than relied upon as a standalone diagnostic biomarker for hvKP. Moreover, this study highlights the crucial need for the development of standardized identification criteria, depending on specific virulence‐associated genes, for particular molecular definitions and detection as well as tracking of the global dissemination of phenotypically unrecognizable hvKP. This identification could consequently help in providing proper insights for infection control and prevention of possible outbreaks of such highly virulent strains. Finally, managing hvKP bacterial diseases needs antimicrobial treatment that is primarily based on the infection site as well as antimicrobial sensitivity testing.

## 5 Limitations

This study provides valuable molecular insights into hvKP determinants and offers a practical, accessible framework for hvKP screening in resource‐limited clinical settings. However, several limitations should be considered when interpreting our findings. First, isolates were collected from a single tertiary‐care hospital, which may limit the generalizability of the prevalence estimates and gene‐association findings to other geographic regions or healthcare settings with different epidemiological profiles. Second, as discussed above, the operational gene‐based definition of hvKP used in this study, while grounded in established genomic frameworks, is not a universally standardized criterion, and classification outcomes may differ if alternative gene combinations or thresholds are applied.

Third, our hvKP classification relied on the presence of specific virulence genes without functional validation of the virulence phenotype, such as serum resistance assays, macrophage killing assays, or *in vivo* virulence models (e.g., Galleria mellonella or murine infection models), which would more directly confirm the hypervirulent phenotype. Fourth, the moderate sample size (82 *K. pneumoniae* isolates: comprising 34 hvKP and 48 cKP) and the wide confidence intervals observed around several diagnostic and association estimates reflect this limited statistical power; larger multicenter cohorts are needed to validate these findings. Finally, this study did not incorporate multilocus sequence typing or whole‐genome sequencing which would have allowed clonal lineage determination, more comprehensive virulence and resistance gene profiling, and identification of the full hvKP‐associated plasmid repertoire beyond the targeted PCR panel used here. To confirm and expand on these results, more research using functional virulence tests, whole‐genome sequencing, and multicenter sampling is necessary.

## Author Contributions

**Fathy M. Elkady:** conceptualization, software, project administration, writing – review and editing. **Nosiba S. Basher:** software, data curation. **Mohammed S. Abdulrahman:** conceptualization, formal analysis, writing – original draft. **Amr H. Hashem:** methodology, investigation, writing – review and editing. **Rafik Abdellatif Metwally:** software, validation, resources. **Ahmed Abdel Tawab:** data curation, formal analysis, writing – original draft. **Ebrahim Saied:** conceptualization, methodology, writing – review and editing. **Nasir A. Ibrahim:** software, validation, resources. **Mohammed Aufy:** validation, formal analysis.

## Ethics Statement

This study was approved by the Scientific Research Ethics Committee, Faculty of Pharmacy, Al‐Azhar University, Cairo, Egypt (Approval No. Azhar‐Pharmacy‐2025‐018). All procedures were conducted in accordance with institutional guidelines and ethical standards.

## Consent

The authors have nothing to report.

## Conflicts of Interest

The authors declare no conflicts of interest.

## Data Availability Statement

All data generated and/or analyzed during the current study are included in this published article. In addition, the complete uncropped agarose gel electrophoresis images supporting this study have been deposited in Figshare and are publicly available at: https://doi.org/10.6084/m9.figshare.31045402.

## Supporting information


Supporting File
