# Supplementary material for: Molecular Insights Into Hypervirulent Klebsiella pneumoniae: Identification and Correlation of Virulence Determinant
Source: Microbiologyopen. 2026 Oct 14;15(5):e70433. doi: 10.1002/mbo3.70433 (PMC13667796; doi:10.1002/mbo3.70433)
Supplement: Supplementary file 1 [file MBO3-15-e70433-s001.docx]

**Figures**

**Figure 1:** Agarose gel electrophoresis showing PCR amplicons of *khe* gene. Lane M: 1500 bp DNA ladder, lane 1: negative control test, lane 2: *khe* gene specific band (about 428 bp) using DNA of *K. pneumoniae* ATCC13882 standard strain as a positive control, lanes 3-10: representative *khe* gene specific bands using DNA of phenotypically detected *K. pneumoniae* isolates 1-8, respectively.

A

B

**Figure 2:** Representative agarose gel of PCR amplicons for different genes. Lanes; M: 1500 bp DNA ladder, 1: negative control, and 2-9: target genes specific bands. (A) lanes 2-3: *rmp*A, lanes 4-5: *rmp*A2, lanes 6-7: *iro*N, and lanes 8-9: *iuc*A. (B) lanes 2-3: *wzi*, lanes 4-5: *wab*G, lanes 6-7: *peg*344 gene, and lanes 8-9: *mag*A.
